# Rapid induction of gliogenesis in OLIG2 and NKX2.2‐expressing progenitors‐derived spheroids

**DOI:** 10.1002/sctm.19-0455

**Published:** 2020-07-27

**Authors:** Wonjin Yun, In Yong Kim, Gwonhwa Song, Seungkwon You

**Affiliations:** ^1^ Laboratory of Cell Function Regulation, Department of Biotechnology, College of Life Sciences and Biotechnology Korea University Seoul South Korea; ^2^ Institute of Animal Molecular Biotechnology Korea University Seoul South Korea; ^3^ Department of Biotechnology, Institute of Animal Molecular Biotechnology Korea University Seoul South Korea

**Keywords:** drug target, embryonic stem cells, glia, induced pluripotent stem cells, oligodendrocytes

## Abstract

Glial cells are crucial for the development of the central nervous system and the maintenance of chemical homeostasis. The process of gliogenesis has been well studied in the rodent brain, but it remains less well studied in the human brain. In addition, rodent glial cells differ from human counterparts in terms of morphologies, functions, and anatomical locations. Cerebral organoids (also referred to as spheroids) derived from human pluripotent stem cells (hPSCs) have been developed and are suitable cell‐based models for researching developmental and neurodegenerative diseases. The in vitro generation of glia, including astrocytes and oligodendrocytes, from such organoids represents a promising tool to model neuronal diseases. Here, we showed that three‐dimensional (3D) culture of OLIG2‐ and NKX2.2‐expressing neurospheres produced efficiently mature astrocytes and oligodendrocytes in terms of morphologies and expression pattern recapitulating native 3D environment. Our findings provide important insights for developmental research of the human brain and glial specification that may facilitate patient‐specific disease modeling.

## Graphical abstract

Human pluripotent stem cell‐derived cerebral organoids can mimic development of the mammalian central nervous system. In this study, we demonstrate that pre‐patterned stem/progenitor cells, especially OLIG2/NKX2.2‐expressing pre‐oligodendrocyte progenitor cells, facilitate glial specification during organoid development. The resulting spheroids can serve as in vitro model for myelination to evaluate promyelination drugs and a source for cell therapy in demyelinating disease.

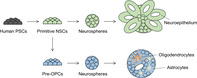

**Significance statement**

This article describes a three‐dimensional (3D) culture system, specifically 3D cerebral organoids (spheroids) that rapidly generate S100β+GFAP+ astrocytes and MBP+ oligodendrocytes recapitulating the developing human brain. The key findings of this study are as follows. (a) Prepatterned stem/progenitor cells, especially OLIG2+NKX2.2+ preoligodendrocyte progenitor cells, facilitate glial specification during organoid development within 8 weeks; (b) the gradual glial specification inside the spheroids and diverse phenotype reflect that in the developing human brain; and (c) the resulting spheroids can serve as in vitro model for myelination to evaluate promyelination drugs such as miconazole and a source for cell therapy in demyelinating disease.

## 1 INTRODUCTION

Over the last several decades, pioneering discoveries have led to a deeper understanding of the roles of macroglia in the central nervous system (CNS).^1^ Astrocytes and oligodendrocytes are two major classes of macroglia derived from neuroepithelial origin.^2^ The star‐shaped cells, astrocytes, contribute to the homeostasis and defense of the CNS through adult neurogenesis.^3^ Oligodendrocytes are the myelinating cells of CNS that enable fast salutatory nerve conduction and provide axonal stability with myelin sheaths.^4^ Glial dysfunction has been involved in demyelinating diseases including multiple sclerosis and amyotrophic lateral sclerosis.^5^, ^6^ Considering heterogeneity of glial cells distributed throughout the CNS^7^ and species differences,^8^ strategic approaches to managing neurodegenerative diseases are needed. Alternatively, human induced pluripotent stem cells (hiPSCs) have been considered as a suitable model for understanding development and neurodegenerative diseases.^9^, ^10^ Indeed, development of cerebral organoids from hiPSCs offers considerable promise as an innovative tool for discovery of therapeutics, thereby paving the road toward personalized medicine.^11^ The in vitro generation of astrocytes^12^, ^13^ and oligodendrocytes^14^, ^15^ in such organoids represents a promising tool to model neuronal diseases but current strategies depend on prolonged three‐dimensional (3D) cultures more than 3 months.

Previously, we established primitive neural stem cells (pNSCs, expressing PAX6 and SOX1) and intermediate progenitor cells (expressing OLIG2 and NKX2.2) from hPSCs.^16^ OLIG2 and NKX2.2‐expressing progenitor cells are known as preoligodendrocyte progenitor cells (pre‐OPCs) which are committed to SOX10 and PDGFRa‐expressing OPCs.^17^ Accordingly, we hypothesized that prepatterned stem/progenitor cells, especially OLIG2 and NKX2.2‐expressing pre‐OPCs, may facilitate glial specification during organoid development.

## 2 MATERIALS AND METHODS

pNSCs and pre‐OPCs were established and cultured as previously described.^16^ The resulting neurospheres (>200 μm) were embedded in Matrigel droplets at day 4 as previously described.^11^ Embedded neurospheres were cultured in appropriate conditions until 8 weeks. For more details, see Supporting Information.

## 3 RESULTS

### 3.1 Rapid neural specification in pre‐OPCs‐derived spheroids

**FIGURE 1:**
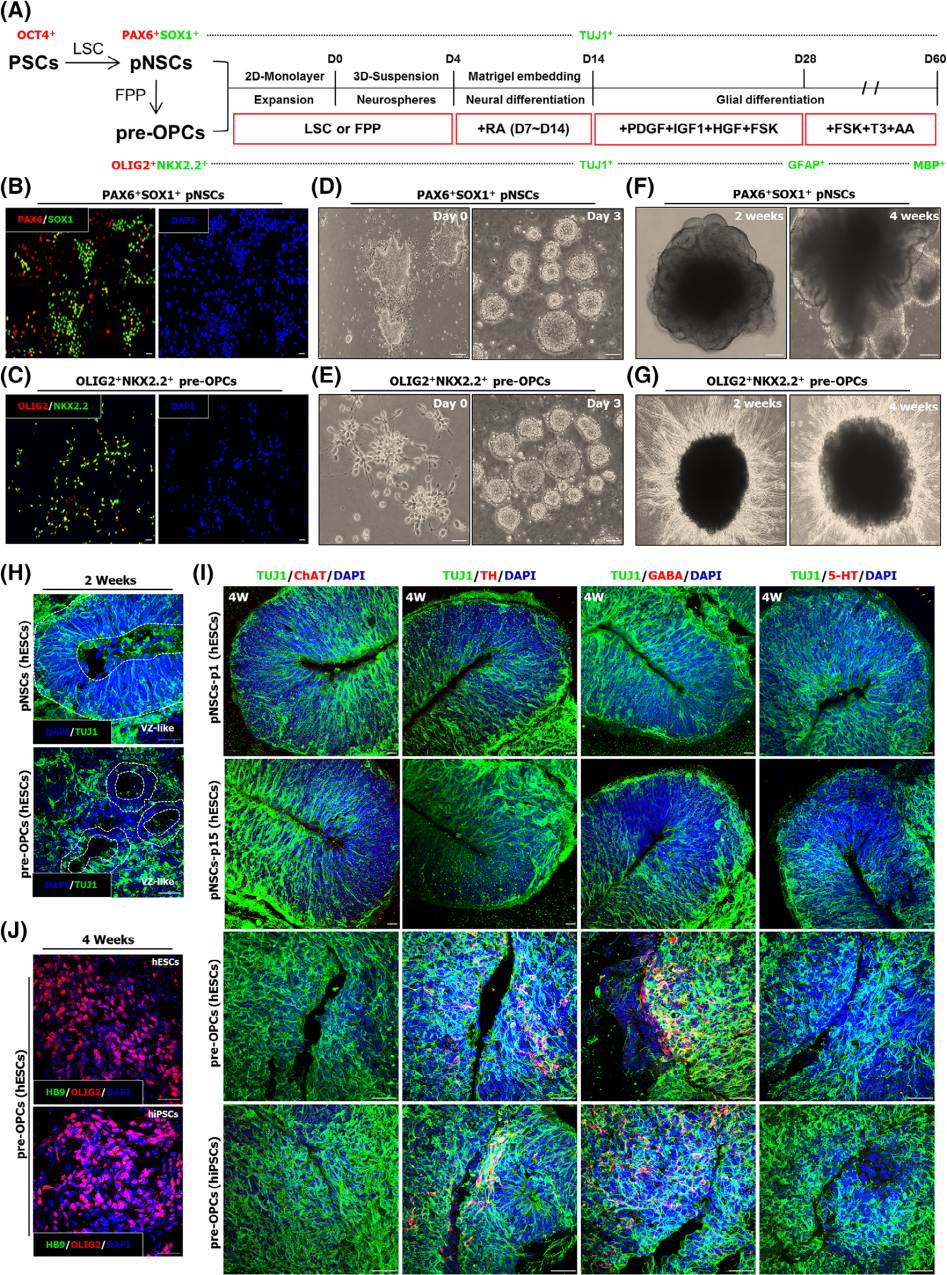
Generation of 3D spheroids using pNSCs and pre‐OPCs. A, Schematic protocol for stepwise differentiation of pNSCs‐ and pre‐OPCs‐derived spheroids into neurons (TUJ1^+^), astrocytes (GFAP^+^), and oligodendrocytes (MBP^+^). LSC: LIF, SB431542, and CHIR99021; FPP: FGF2, PDGF‐AA, and Purmorphamine; FSK: Forskolin; T3: 3,3′,5‐triiodo‐l‐thyronine; AA: ascorbic acid. B,C, Characterization of pNSCs (B) and pre‐OPCs (C) by immunofluorescence staining for PAX6, SOX1, OLIG2, and NKX2.2. Scale bars = 100 μm. D,E, Representative phase contrast images of pNSCs (D) and pre‐OPCs (E) on day 0 and 3. Scale bars = 100 μm. F,G, Time course images showing 4 weeks of differentiation inside the Matrigel droplets. Before embedding, pNSCs and pre‐OPCs formed neurospheres 200 to 300 μm in diameter. Scale bars = 500 μm. H, Representative fluorescence images of cryosections 2 weeks after Matrigel embedding. Dashed lines indicate ventricular like‐zones (VZ‐like). Scale bars = 30 μm. I,J, Representative fluorescence images of cryosections of pNSCs (passage 1 and passage 15)‐ and pre‐OPCs‐derived spheroids 4 weeks after Matrigel embedding. Scale bars = 30 μm. 5‐HT, 5‐hydroxytryptamine; ChAT, choline acetyltransferase; HB9, MNX1; OPC, oligodendrocyte progenitor cell; pNSC, primitive neural stem cell; TH, tyrosine hydroxylase

For in vitro glia model, PAX6 and SOX1‐expressing pNSCs or OLIG2 and NKX2.2‐expressing pre‐OPCs were prepared based on recently established protocols for generating cerebral organoids,^11^, ^15^ which includes exposure to retinoic acid for 1 week and further treated with PDGF‐AA, IGF‐1, HGF, and forskolin for 2 weeks. Afterward, these cells were cultured for up to 4 weeks in glial differentiation medium containing forskolin, T3 (3,3′,5‐triiodo‐l‐thyronine), and ascorbic acid (Figure 1A). As previously observed, characterization of pNSCs and pre‐OPCs was assessed by immunocytochemistry (Figure 1B,C); these two types of cells easily formed neurospheres (Figure 1D,E), embedded into Matrigel droplets. After 7 days of exposure to retinoic acid (on day 14), pNSCs‐derived spheroids allowed the formation of large neuroepithelial buds as observed in cerebral organoids,^11^ whereas radially protruding processes consistent with direct neural differentiation were observed in pre‐OPCs‐derived spheroids (Figure 1F,G). Immunofluorescence staining of cryosectioned spheroids revealed that pNSCs‐derived spheroids have a large ventricular like‐zones, containing TUJ1^+^ immature neurons, while pre‐OPCs‐derived spheroids formed a small ventricular like‐zones at week 2 of culture (Figure 1H). Further characterization showed that the vast majority of neurons in pNSCs (passage 1 and passage 15)‐derived spheroids remained immature (TUJ1^+^), organized into ventricular like‐zones, while pre‐OPCs‐derived spheroids were composed of dopaminergic neurons (TH^+^) and GABAergic neurons (GABA^+^), but not cholinergic neurons (ChAT^+^) and serotonin neurons (5‐HT^+^) (Figure 1I). Interestingly, even though pre‐OPCs‐derived spheroids remained to express OLIG2, a marker for the motor neuron,^18^ we could not observe HB9^+^ motor neurons at week 4 of differentiation (Figure 1J). Similarly, this may result from FGF2 culture to establish pre‐OPCs blocked HB9 (MNX1) expression as previously described.^17^ Thus, our finding indicates that TUJ1^+^ immature neurons in pre‐OPCs‐derived spheroids were rapidly specified into functional neurons at the expense of organizing early developing cortex such as ventricular zone.

### 3.2 Rapid glial specification in pre‐OPCs‐derived spheroids

**FIGURE 2:**
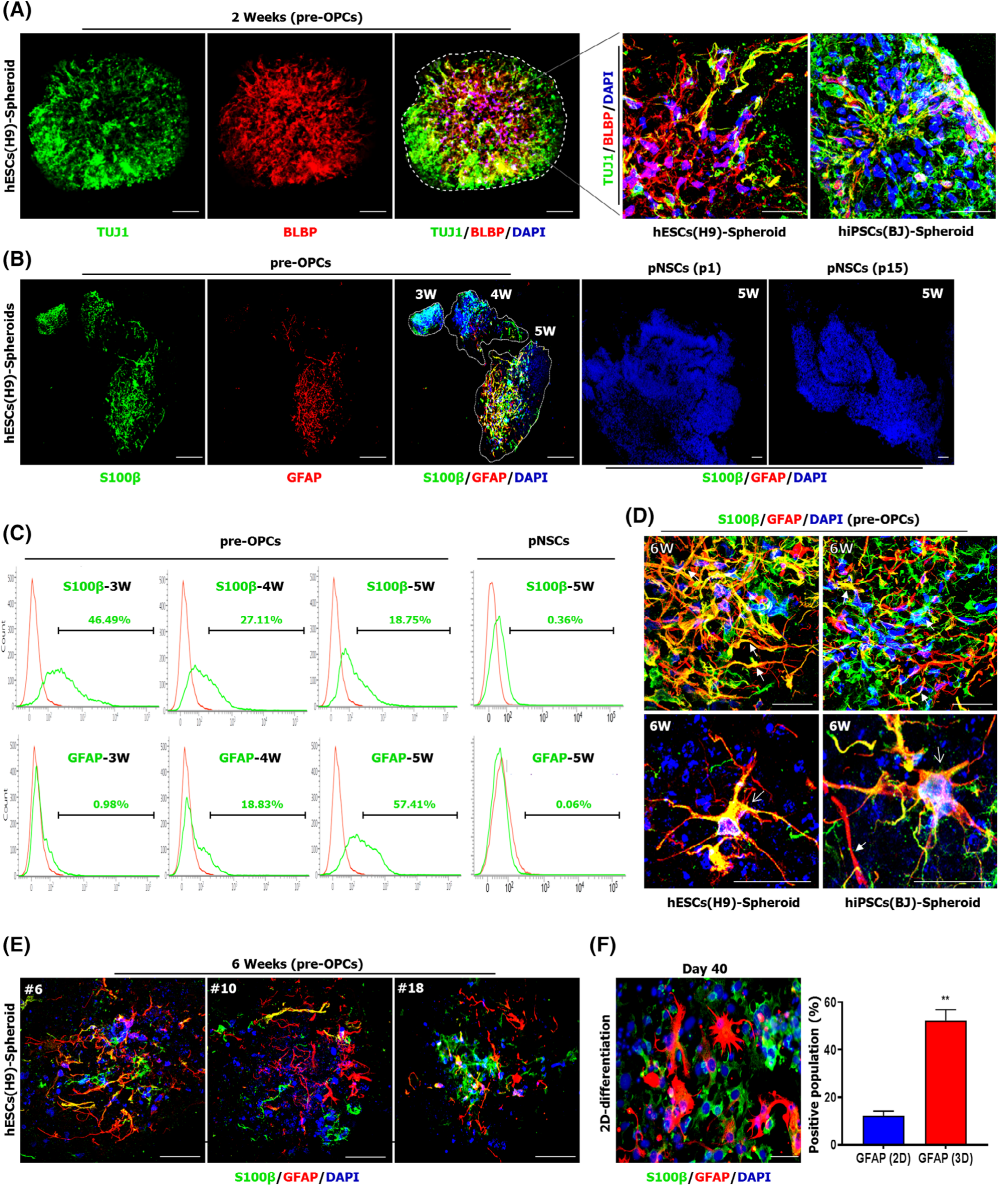
Spheroids culture of pre‐OPCs enables rapid production of astrocytes. A, Representative fluorescence images of cryosectioned pre‐OPCs‐derived spheroids stained for TUJ1 (green) and brain lipid binding protein (BLBP; red). Scale bars = 100 μm (left panel), 30 μm (right panel). B, Representative fluorescence images of cryosections after Matrigel embedding. Dashed lines indicate separated spheroids. Cryosectioned spheroids were stained for S100β (green) and GFAP (red). Scale bars = 200 μm. C, Representative time course flow cytometry data showing S100β‐ or GFAP‐positive populations. Data from six (three each for H9‐hESCs and hiPSCs) independent experiments are presented as mean values. D, Higher magnification fluorescence image of 4‐week‐old and 6‐week‐old pre‐OPCs‐derived spheroids. The arrows denote elongated shaped astrocytes and arrowheads denote star shaped astrocytes. Scale bars = 30 μm. E, Higher magnification fluorescence image of 6‐week‐old pre‐OPCs‐derived spheroids derived from H9‐hESCs (nos. 6 and 10) and hiPSCs (nos. 18). Scale bars = 30 μm. F, Representative fluorescence image of monolayer differentiation: GFAP (red) and S100β (green). Comparable flow cytometry data (2D vs 3D) from three independent experiments are presented. hiPSC, human induced pluripotent stem cell; OPC, oligodendrocyte progenitor cell; pNSC, primitive neural stem cell. **, *P* < .01

Next, we investigated whether these pre‐OPCs‐derived spheroids allow rapid initiation of glial specification. We observed that radially extended TUJ1^+^ neurons were followed by BLBP‐positive radial glial cells (or glial‐restricted progenitors) in pre‐OPCs‐derived spheroids (Figure 2A), suggesting that glial differentiation had progressed inside the spheroids. S100β^+^ or GFAP^+^ populations were detectable from 3 weeks in pre‐OPCs‐derived spheroids whereas those markers were barely detectable in pNSCs‐derived spheroids at week 4 (Figure 2B,C). Immunostaining revealed that elongated and star‐shaped astrocytes were detectable at week 6 (Figure 2D), implying morphologically diverse progeny reflecting the developmental process.^19^ Interestingly, we observed single‐positive (S100β or GFAP) populations in most of the spheroids after 6 weeks in culture (Figure 2E), observed in separate cerebral regions.^20^ Furthermore, this 3D culture approach contributed to rapid and efficient production of GFAP^+^ astrocytes compared to our previous monolayer culture^16^ (Figure 2F). These findings suggest that pre‐OPCs‐derived spheroids allow development of morphologically and immunologically diverse astrocytes in a rapid and efficient manner.

**FIGURE 3:**
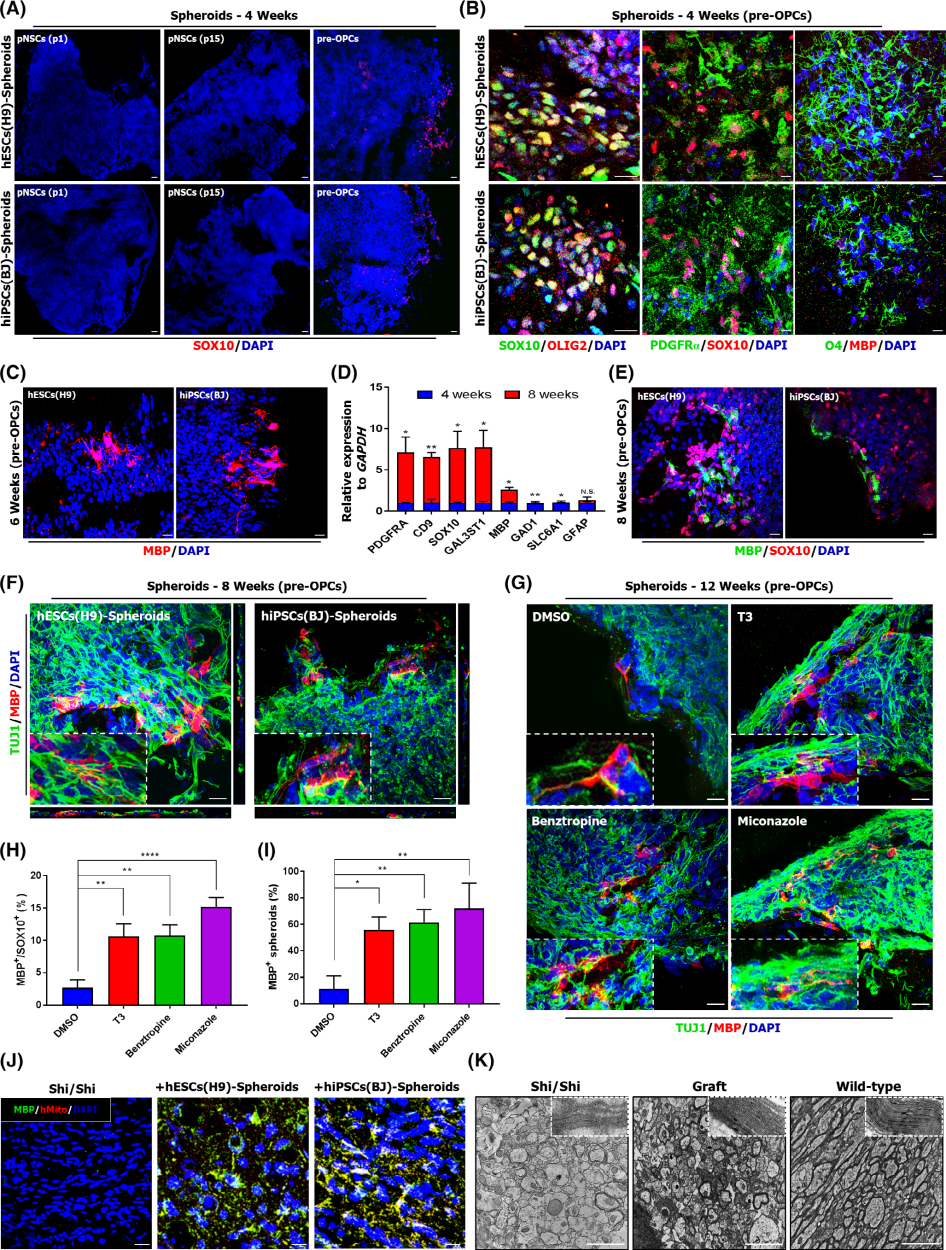
Spheroids culture of pre‐OPCs enables rapid production of oligodendrocytes. A, Representative fluorescence images of cryosectioned spheroids stained for SOX10 (red) 4 weeks after Matrigel embedding. Scale bars = 200 μm. B,C, Higher magnification fluorescence image of 4‐week‐old (B) and 6‐week‐old (C) pre‐OPCs‐derived spheroids. Scale bars = 20 μm. D, quantitative polymerase chain reaction (qPCR) analysis for oligodendrocyte markers in 4‐week‐old and 8‐week‐old pre‐OPCs‐derived spheroids. Expression levels are shown relative to levels in 4‐week‐old spheroids and normalized to *GAPDH* expression. Data are represented as means + SDs (n = 3). E,F, Higher magnification fluorescence image of 8‐week‐old pre‐OPCs‐derived spheroids stained for MBP, SOX10, or TUJ1. Scale bars = 20 μm. G, Representative fluorescence images of dimethyl sulfoxide (DMSO), T3, Benztropine, or Miconazole‐treated pre‐OPCs‐derived spheroids stained for TUJ1 and MBP after 12 weeks culture. Scale bars = 20 μm. H,I, Quantification of MBP^+^ populations among SOX10^+^ populations (H) and MBP^+^ spheroids among the whole spheroids (I) after treatment with either DMSO, T3, Benztropine, or Miconazole. Data from three independent experiments are presented as means + SD. J, Transplantation of magnetic‐activated cell sorting (MACS)‐purified O4^+^ oligodendrocytes from Miconazole‐treated spheroids into shiverer mice. Scale bars = 20 μm. K, Representative electron microscopic images of compact myelin. Samples of engrafted brains were obtained 8 weeks after engraftment. Scale bars = 2 μm. OPC, oligodendrocyte progenitor cell; pNSC, primitive neural stem cell. *, *P* < .05; **, *P* < .01; ****, *P* < .0001

Meanwhile, we observed some populations positive for SOX10 localized to the outer surface of pre‐OPCs‐derived spheroids at week 4 (Figure 3A) and these SOX10^+^ cells also expressed OLIG2 and PDGFRα (Figure 3B). These cells acquired the phenotype of O4^+^ immature oligodendrocytes (MBP at a low level) at week 4 (Figure 3B) and MBP^+^ oligodendrocytes at week 6 (Figure 3C). Upon further culturing, several oligodendrocyte markers, including CD9, GAL3ST1, and MBP, were increased in 8‐week‐old pre‐OPCs‐derived spheroids (Figure 3D) and these oligodendrocytes were capable of myelinating with TUJ1^+^ neurons (Figure 3E,F). Next, to evaluate whether pre‐OPCs‐derived spheroids can serve as in vitro model for myelination, we treated promyelination drugs, such as Benztropine and Miconazole, which have exhibited drug‐mediated remyelination in vivo^21^, ^22^ from week 4 to 12. Notably, at 8 weeks of spheroid culture with promyelination drugs, robust populations of myelinating oligodendrocytes were generated when compared to DMSO‐treated spheroids (Figure 3G). While benztropine‐treated spheroids showed similar results to T3‐treated spheroids, miconazole‐treated spheroids exhibited elevated MBP^+^/SOX10^+^ populations (15.18%) and abundant MBP^+^ spheroids (n = 14/18) (Figure 3H,I). These results were consistent with previous study showing that miconazole‐treatment resulted in robust generation of MBP^+^ cells.^23^ Furthermore, MACS‐purified O4^+^ oligodendrocytes in miconazole‐treated spheroids were engrafted into MBP‐deficient shiverer mice by the method, as previously reported^16^ and these transplanted cells resulted in MBP^+^ oligodendrocytes (Figure 3J) as well as contributed to myelin compaction after 12 weeks of transplantation, detected by electron microscopy (Figure 3K).

## 4 DISCUSSION

**FIGURE 4:**
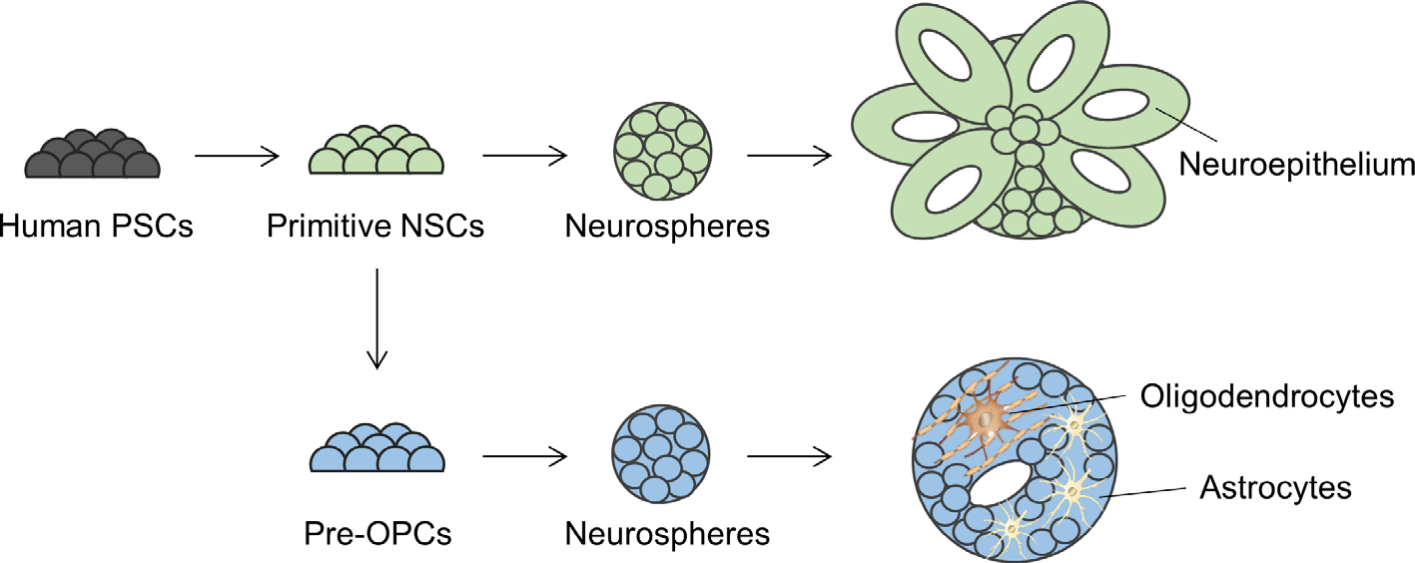
Schematic summary of the present study. NSC, neural stem cell; OPC, oligodendrocyte progenitor cell; PSC, pluripotent stem cell

The present study demonstrated that our spheroid culture is worthy of consideration as an in vitro screening tool for evaluation of drug candidates in myelin/oligodendrocyte‐related dysfunction in a time‐ and cost‐effective and patient‐specific manner. In the developing CNS, the neurogenesis‐to‐gliogenesis switch is mediated by spatiotemporal regulation of gene expression such as OLIG2 and NKX2.2 which help orchestrate transition from neurogenic to gliogenic. In the present study, OLIG2 and NKX2.2‐expressing neurospheres exhibited neurogenesis in response to retinoic acids and then glial specification progressed rapidly after the protrusion of neural cells from the spheroids. Spheroid culture starting from intermediate stage or precommitted stem/progenitor cells may facilitate the production of astrocytes and oligodendrocytes (Figure 4). Moreover, these OLIG2 and NKX2.2‐expressing pre‐OPCs could be propagated for 20 passages,^16^ with repeated freeze‐thaw cycles, and continued to robustly express OLIG2 and NKX2.2, allowing reproducibility and repeatability by reduced batch‐to‐batch variability. Understanding the heterogeneity of inherent glial cells in terms of morphological, functional, and anatomical variations depending on different brain regions remains largely unknown.^7^, ^24^, ^25^ Thus, multidimensional organized‐models reflecting pathological brain regions are required for better understanding of human glial cells and their further applications.

## 5 CONCLUSION

In summary, our culture system would be useful and valuable for deeper understanding of glial development and as an in vitro platform to explore the cellular and molecular cues that govern neurodegenerative and neuropsychiatric impairments.

## CONFLICT OF INTEREST

The authors declared no potential conflicts of interest.

## AUTHOR CONTRIBUTIONS

W.Y.: conception and design, collection and/or assembly of data, manuscript writing; I.Y.K.: collection and/or assembly of data, manuscript writing; G.S.: financial support, provision of study material or patients, data analysis and interpretation; S.Y.: conception and design, administrative support, final approval of manuscript.

## Supporting information


**Table S1** Sequences of the primers used for RT‐PCR and qPCR.
**Table S2**. List of Primary Antibodies.
**Table S3**. List of Secondary Antibodies.

## Data Availability

The data that support the findings of this study are available on request from the corresponding author.
