# Supplementary material for: Rapid induction of gliogenesis in OLIG2 and NKX2.2‐expressing progenitors‐derived spheroids
Source: Stem Cells Transl Med. 2020 Jul 27;9(12):1643–50. doi: 10.1002/sctm.19-0455 (PMC7695630; doi:10.1002/sctm.19-0455)
Supplement: Supplementary file 1 — Table S1 Sequences of the primers used for RT‐PCR and qPCR. Table S2. List of Primary Antibodies. Table S3. List of Secondary Antibodies. [file SCT3-9-1643-s001.docx]

**Supporting Material and Methods**

1. Cell culture

The pNSCs (expressing PAX6 and SOX1) and pre-OPCs (intermediate progenitor cells, expressing OLIG2 and NKX2.2) were established and cultured as previously described [16]. Briefly, before Matrigel (356231, Corning, NY, USA) embedding, pNSCs were cultured in LSC medium (containing LIF, SB431542, and CHIR99021) and pre-OPCs were cultured in FPP medium (containing FGF2, PDGF-AA, and Purmorphamine). Considering age of culture, pNSCs (passage 15, close to pre-OPCs stage) were also prepared. Cells were dispersed at day 0 with Accutase (EMD Millipore, Burlington, MA, USA), seeded on ultra-low-attachment plates (Corning), and maintained in LSC or FPP medium until embedding. The resulting neurospheres (>200 µm) were embedded in Matrigel droplets at day 4 as previously described [11]. Embedded neurospheres were cultured in the Basal medium consisting of DMEM/F12 (Thermo Fisher Scientific, Waltham, MA, USA) supplemented with 543 μg/ml sodium bicarbonate (Sigma-Aldrich, St. Louis, MO, USA), 1× N-2 supplement (Thermo Fisher Scientific), 1× B-27 supplement without vitamin A (Thermo Fisher Scientific), 1% penicillin–streptomycin, 1% l-glutamine (Thermo Fisher Scientific), 1% nonessential amino acids (Thermo Fisher Scientific), 5 μg/ml insulin (Sigma-Aldrich) for three days. Then, the droplets were transferred to the orbital shaker and cultured in Basal medium supplemented with 0.5 μM retinoic acid (Sigma-Aldrich) for additional 1 week, and the medium was switched to Basal medium supplemented with 10 ng/ml PDGF-AA (Peprotech), 10 ng/ml IGF-1 (Peprotech), 10 ng/ml NT-3 (Peprotech), 5 ng/ml HGF (Peprotech), and 10 μM forskolin (Tocris) for two weeks and then the droplets were cultured in the glial differentiation medium consisting of Basal medium, 10 μM forskolin (Tocris), 60 ng/ml 3,3′,5-triiodo-l-thyronine (T3, Sigma-Aldrich), and 20 μg/ml ascorbic acid (Peprotech) for four weeks. For promyelinating compound screen, 4-week-old spheroids were treated with DMSO (Sigma-Aldrich), 60 ng/ml T3 (Sigma-Aldrich), 1 μM benztropine (Sigma-Aldrich), or 1 μM miconazole (Sigma-Aldrich) for eight weeks.

2. Sample preparation and analysis

Spheroid samples were embedded in O.C.T. compound (Tissue Tek; Sakura Finetek USA, Inc., Torrance, CA, USA) and cryosectioned at a thickness of 20 μm. Sections were blocked with 2% normal donkey serum in PBS with 0.2% Triton X-100 for 1 h, incubated overnight at 4°C with the designated primary antibodies (see Supplementary Tables S2 and S3), and then incubated at room temperature for 1 h with the appropriate Alexa Fluor 488- or 594-conjugated secondary antibodies (Thermo Fisher Scientific). Nuclei were counterstained with 1 μg/ml DAPI (Sigma-Aldrich) for 5 min. The samples were rinsed three times with PBS and observed under an Olympus confocal laser scanning microscope. Preparations of engrafted mouse brains and TEM analysis were performed as previously described [16].

3. Flow cytometry

Spheroids were mechanically dissociated and harvested with Accutase, rinsed three times with cold PBS, blocked with 5% normal donkey serum for 15 min, and incubated first with primary antibodies against S100β and GFAP for 30 min and then with Alexa Fluor-conjugated secondary antibodies for 30 min. After staining, the cells were fixed with 0.5% formaldehyde. Cells were permeabilized with 0.1% Triton X-100 during the blocking process. The cells were then analyzed by flow cytometry on a FACS Verse flow cytometer (BD Biosciences, San Jose, CA, USA).

4. qPCR analysis

Quantitative PCR (qPCR) was performed on cDNA in triplicate reactions. The negative controls included a reverse transcription-negative blank of each sample and a no-template blank. Gene expression levels were normalized against the corresponding level of *GAPDH*, which was used as an internal control. The primers used for qPCR are listed in Supplementary Table S1.

5. Statistical analysis

The data are shown as the means ± standard deviations (SDs) from three to six replicates. Data were analyzed by unpaired two-tailed Student’s *t* tests and analysis of variance (ANOVA). A *P* value of <0.05 was considered statistically significant. *, *P* < 0.05; **, *P* < 0.01; ***, *P* < 0.001; ****, *P* < 0.0001.

**Table S1. Sequences of the primers used for RT-PCR and qPCR.**

| **Genes** | **Primer sequences (5’ to 3’)** | **Type** |
| --- | --- | --- |
| PDGFRα | CAGTCCTGGTGCTGTTGGTG | Forward |
|  | CCGGCTTAATCCATAGGCTG | Reverse |
| SOX10 | AAGCCCAGGTGAAGACAGAGAC | Forward |
|  | CCATATAGGAGAAGGCCGAGTAGA | Reverse |
| MBP | CCAGAGCAGCCTCTATGAACAA | Forward |
|  | GGAAGTGAATGAGCCGGTTATC | Reverse |
| GFAP | GGCAACTCGTACCCAAATTTCCAA | Forward |
|  | TCACACGATTCTCCATCATCCTCC | Reverse |
| FABP7 | GATTTTTGCCCACCCTCTTTCCAA | Forward |
|  | CGCCTAGAGCCTTCATGTACTCAT | Reverse |
| SOX9 | GGCAACTCGTACCCAAATTTCCAA | Forward |
|  | CGCCTAGAGCCTTCATGTACTCAT | Reverse |

**Table S2. List of Primary Antibodies.**

| **Primary antibody** | **Dilution** | **Host** | **Provider** |
| --- | --- | --- | --- |
| PAX6 | 1:100 | Mouse | DSHB (PAX6) |
| SOX1 | 1:100 | Goat | R&D Systems (AF3369) |
| OLIG2 | 1:200 | Rabbit | Merck Millipore (AB9610) |
| NKX2.2 | 1:100 | Mouse | DSHB (74.5A5) |
| TUJ1 | 1:1000 | Mouse | BioLegend (801202) |
| GABA | 1:200 | Rabbit | Sigma (A2052) |
| TH | 1:200 | Rabbit | Merck Millipore (AB152) |
| 5-HT | 1:5000 | Rabbit | Immunostar (20080) |
| HB9 | 1:100 | Mouse | DSHB (81.5C10) |
| ChAT | 1:500 | Goat | Merck Millipore (AB144P) |
| BLBP | 1:200 | Rabbit | Merck Millipore (ABN14) |
| PDGFRα | 1:200 | Rabbit | Santacruz (SC-338) |
| GFAP | 1:500 | Rabbit | Merck Millipore (AB5804) |
| S100β | 1:500 | Mouse | SIGMA-ALDRICH (S2532) |
| O4 | 1:200 | Mouse | R&D Systems (AB1326) |
| MBP | 1:50 | Rat | Abcam (AB7349) |
| hMitochondria | 1:100 | Mouse | Merck Millipore (MAB1273) |

**Table S3. List of Secondary Antibodies.**

| **Secondary antibody** | **Dilution** | **Host** | **Provider** |
| --- | --- | --- | --- |
| Alexa Fluor 488 (Mouse IgG) | 1:500 | Donkey | Thermo Fisher (A-21202) |
| Alexa Fluor 488 (Rabbit IgG) | 1:500 | Donkey | Thermo Fisher (A-21206) |
| Alexa Fluor 488 (Goat IgG) | 1:500 | Donkey | Thermo Fisher (A-11055) |
| Alexa Fluor 488 (Mouse IgM) | 1:500 | Goat | Thermo Fisher (A-21042) |
| Alexa Fluor 488 (Rat IgG) | 1:500 | Donkey | Thermo Fisher (A-21208) |
| Alexa Fluor 594 (Mouse IgG) | 1:500 | Donkey | Thermo Fisher (A-21203) |
| Alexa Fluor 594 (Rabbit IgG) | 1:500 | Donkey | Thermo Fisher (A-21207) |
| Alexa Fluor 594 (Goat IgG) | 1:500 | Donkey | Thermo Fisher (A-11058) |
| Alexa Fluor 594 (Rat IgG) | 1:500 | Donkey | Thermo Fisher (A-21209) |
